# Increased B Cell and Cytotoxic NK Cell Proportions and Increased T Cell Responsiveness in Blood of Natalizumab-Treated Multiple Sclerosis Patients

**DOI:** 10.1371/journal.pone.0081685

**Published:** 2013-12-02

**Authors:** Johan Mellergård, Måns Edström, Maria C. Jenmalm, Charlotte Dahle, Magnus Vrethem, Jan Ernerudh

**Affiliations:** 1 Neurology, Department of Clinical and Experimental Medicine, Faculty of Health Sciences, Linköping University, and Department of Neurology, County Council of Östergötland, Linköping, Sweden; 2 Clinical Immunology, Unit of Autoimmunity and Immune Regulation, Department of Clinical and Experimental Medicine, Faculty of Health Sciences, Linköping University, Linköping, Sweden; 3 Department of Clinical Immunology and Transfusion Medicine, County Council of Östergötland, Linköping, Sweden; 4 Neurology and Clinical Neurophysiology, Department of Clinical and Experimental Medicine, Faculty of Health Sciences, Linköping University, and Department of Neurology and Neurophysiology, County Council of Östergötland, Linköping, Sweden; Innsbruck Medical University, Austria

## Abstract

**Background:**

Changes in the blood lymphocyte composition probably both mediate and reflect the effects of natalizumab treatment in multiple sclerosis, with implications for treatment benefits and risks.

**Methods:**

A broad panel of markers for lymphocyte populations, including states of activation and co-stimulation, as well as functional T cell responses to recall antigens and mitogens, were assessed by flow cytometry in 40 patients with relapsing multiple sclerosis before and after one-year natalizumab treatment.

**Results:**

Absolute numbers of all major lymphocyte populations increased after treatment, most markedly for NK and B cells. The fraction of both memory and presumed regulatory B cell subsets increased, as did CD3^-^CD56^dim^ cytotoxic NK cells, whereas CD3^-^CD56^bright^ regulatory NK cells decreased. The increase in cell numbers was further associated with a restored T cell responsiveness to recall antigens and mitogens in functional assays.

**Conclusions:**

Our data confirms that natalizumab treatment increases the number of lymphocytes in blood, likely mirroring the expression of VLA-4 being highest on NK and B cells. This finding supports reduction of lymphocyte extravasation as a main mode of action, although the differential effects on subpopulation composition suggests that cell-signalling may also be affected. The systemic increase in T cell responsiveness reflects the increase in numbers, and while augmenting anti-infectious responses systemically, localized responses may become correspondingly decreased.

## Introduction

The pathogenesis of multiple sclerosis (MS) has been linked to T cells-mediated immune regulation, involving both CD4^+^ T helper and CD8^+^ T cytotoxic cells [1]. However, the pathogenic scenario has become more diverse including B cells [2,3], dendritic cells, natural killer (NK) cells and T cells with NK cell properties (NKT) [4]. Natalizumab, a humanized monoclonal antibody approved for the treatment of relapsing MS, is directed against the α4-chain of VLA-4 (α4β1) and α4β7 integrins present on lymphocytes. Natalizumab blocks the binding between these integrins and their endothelial receptors, vascular cell adhesion molecule-1 (VCAM-1) and mucosal addressin-cell adhesion molecule 1 (MadCAM-1) [5]. Consequently, treatment leads to a decline in the migration of potentially disease-promoting lymphocytes into the central nervous system (CNS), resulting in reduced intrathecal inflammation [6-8] and improvement in magnetic resonance imaging (MRI) measurements [9]. As a result of the decreased extravasation, a systemic accumulation of circulating NK cells [10], B cells [11] and pro-inflammatory T cells [12] has been observed after natalizumab treatment. In addition to reduced extravasation of lymphocytes and given the central role of integrins in cell-cell interactions, other immunomodulating mechanisms [13,14] probably contribute to the treatment outcome, including benefits and risks.

Since the first cases of progressive multifocal leukoencephalopathy (PML) in natalizumab-treated patients, it has been debated whether this JC-virus infection is merely a result of reduced immune surveillance in the CNS, or if other treatment mechanisms affecting lymphocyte populations may contribute. To further elucidate the mechanisms of treatment, the effects on lymphocyte populations have been investigated. However, several earlier reports were based on limited patient numbers and focused on restricted and specific blood lymphocyte populations such as CD4^+^ and CD8^+^ T cells [15-17], regulatory T cells (Treg) [18] and B cells [11,17], but did not address the simultaneous effects of natalizumab treatment on a broader panel of different lymphocyte populations and their expression of activation and co-stimulation markers. Furthermore, treatment effects as to functional capacity of lymphocytes have not previously been evaluated longitudinally in patients with MS.

We longitudinally followed 40 patients with MS before and after one-year natalizumab treatment, examining the numbers and proportions of circulating CD4^+^ and CD8^+^ T cells, Treg cells, B cells, NK cells, NKT cells as well as markers of activation and co-stimulation. In addition, functional studies of T cell responses to recall antigens and mitogens were performed. The aims were to explore changes in circulating lymphocyte subpopulation compositions and to assess the functional capacity of T cell responses during natalizumab treatment.

## Methods

### Ethics statement

The study was based on written informed consent, and approved by The Regional Ethics Committee in Linköping (Dnr M180-07 T130-09).

### Patients and controls

Natalizumab treatment (300 mg once a month) was initiated in 40 patients with MS (Table 1). Initiation of treatment was based on clinical and MRI parameters, suggesting an active relapsing disease. All included patients fulfilled the McDonald criteria of MS [19] and were consecutively recruited from the Department of Neurology at the University Hospital, Linköping. Sampling of peripheral blood was obtained before (median 0.75 months, range 0-5.0) and after one year (median 12.0 months, range 10-17) of treatment. Definition of Expanded Disability Status Scale (EDSS) [20] score and Multiple Sclerosis Severity Score (MSSS) [21] were done by a neurologist (CD, MV or JM). The Symbol Digit Modalities Test (SDMT) [22] and the Multiple Sclerosis Impact Scale (MSIS-29) [23] were also performed. In the lymphocyte activation assay (see below) personnel (n=23) at the Department of Clinical immunology and transfusion medicine were recruited as controls, median age 45 years (range 35-59), 21 women and 2 men. All controls were healthy and without drug therapy.

**Table 1:** Patient demographics and disease characteristics at baseline.

Number of subjects	40
Median age (years)	36.5 (range 22-62)
Sex (M/F)	22/18
Median disease duration (years) **^*a*^**	9.5 (range 0.9-30.0)
Diagnosis (RRMS / PRMS)	34/6
EDSS (no. of subjects)	
0-3.5	32
4.0-5.5	4
6.0-7.0	4
Median EDSS	2.5 (range 0-7.0)
Median MSSS	3.82 (range 0.19-8.55)
Treatment **^*b*^**	
Interferon-β	25
Glatiramer acetate	4
IVIG	1
Corticosteroids **^*c*^**	3
No treatment	10
Median number of relapses last two years	2.0 (range 0-8)
Number of patients with relapse last two months	10

*a* Median number of years from first symptoms of MS to inclusion.

*b* Treatment within 4 months before inclusion.

*c* Three patients were treated with high-dose corticosteroids due to relapse, in addition to interferon-β (1 patient) and glatiramer acetate (2 patients) respectively.

Abbreviations: RRMS=relapsing-remitting MS, PRMS=progressive MS with superimposed relapses, EDSS=Expanded Disability Status Scale, IVIG=Intravenous Immunoglobulins

### Flow cytometry

Whole blood was drawn in EDTA tubes. FACS Lysing Solution (BD Biosciences, San José, CA) was added for removal of erythrocytes. In total, five tubes were used, with the following antibody combinations. Tube A; anti-CD45-PerCP, anti-CD3-FITC, anti-CD4-PE-Cy7, anti-CD8-APC-Cy7, anti-CD16/56-PE,anti-CD19-APC, tube B; anti-CD3-PerCP, anti-CD4-PE-Cy7, anti-CD8-APC-Cy7, anti-CD28-PE, anti-CD56-APC, anti-CD57-FITC, tube C; anti-CD3-PerCP, anti-CD4-PE-Cy7, anti-CD8-APC-Cy7, anti-CD56-APC, anti-HLA-DR-FITC, anti-CD69-PE, tube D; anti-CD3-PerCP, anti-CD4-PE-Cy7, anti-CD8-APC-Cy7, anti-CD25-FITC, anti-OX40L-PE, tube E; anti-CD45-FITC, anti-CD19-PE-Cy7, anti-CD5-PerCP-Cy5.5, anti-CD25-APC, anti-CD27-PE and anti-HLA-DR-APC-Cy7 (all antibodies from BD Biosciences). For analysis of absolute cell numbers, Truecount^TM^ tubes (BD Biosciences) were used for tube A. Collection of data was performed using a FACS Canto II with the FACS Diva software (BD Biosciences). Data was analyzed using the Kaluza software v 1.1 (Beckman Coulter, Brea, CA). 

Lymphocytes were gated through forward and side scatter properties, in tubes A and E with the support of a CD45^+^ gate. Populations were defined on the basis of forming discrete populations or by using other populations as negative or positive populations. In tube A, T cells were selected through CD3^+^ expression, and thereafter gated for CD4 and CD8 expression. NK cells (CD16/56^+^) and B cells (CD19^+^) were gated from the CD3^-^ population. For tube B, C and D, CD3^+^ T cells were divided into CD4^+^ and CD8^+^ populations. In tube B, the negative gate for CD28, defining CD4^+^CD28^–^ and CD8^+^CD28^–^ cells, were set through CD28 expression on CD3^–^ cells, and included cells were analyzed for CD56/CD57 expression. For tube C, CD3^–^CD56^+^ NK cells were further gated as CD3^–^CD56^bright^ or CD3^–^CD56^dim^. CD3^–^CD56^+^, CD3^+^CD4^+^ and CD3^+^CD8^+^ cells were analyzed for CD69 and HLA-DR expression. Tube D CD3^+^CD4^+^ and CD3^+^CD8^+^ cells were analyzed for CD25 and OX40L expression. In addition, regulatory (CD3^+^)CD4^dim^CD25^bright^ [24] T cells were analyzed. Finally, in tube E, CD19^+^ B cells were gated for CD25 and CD27 expression, respectively. Polymorphonuclear cells were used to set the gate for CD25^+^ and CD27^+^ B cells.

### Lymphocyte activation assay

To evaluate lymphocyte function we used, with some modifications, the previously described FASCIA method [25]. Briefly, peripheral blood was drawn in Heparin tubes; 50 µL were diluted 1:10 in culturing media, consisting of RPMI 1640 (Gibco BRL, Paisley, Scotland, UK) supplemented with L-glutamine 584 µg/mL (Sigma Aldrich, Stockholm, Sweden), penicillin 200 IE/mL and streptomycin 200 µg/mL (both from Cambrex, New Jersey, USA). Cultures were stimulated with influenza antigen 1:1000 (Vaxigrip; Sanofi Pasteur, Solna, Sweden), purified protein derivate (PPD) 10 µg/mL (SSI, Copenhagen, Denmark), a mix of cytomegalovirus (CMV) peptides 0.125 µg (BD Biosciences), tetanus toxin 5.7 Lf/mL (SSI), phytohaemagglutinin (PHA) 5 µg/mL (Sigma Aldrich), pokeweed mitogen (PWM) 10 µg/mL (Sigma Aldrich) or myelin basic protein (MBP) 100 µg/mL (Sigma Aldrich). Negative controls without antigen were cultured separately. Culturing ensued for seven days at 37°C with 5% CO_2_, after which cells were harvested and labeled with anti-CD3-FITC, anti-CD4-PerCP, anti-CD8-APC and anti-CD108-PE. After labeling, erythrocytes were lysed by incubating cells with 0.8% NH_4_Cl. Collection and analyses of data were performed using a FACS Canto II system running the FACS Diva software. 

First, lymphocyte and lymphoblast gates were set using unstimulated samples (RPMI). The numbers of cells were calculated using Truecount^TM^ tubes. Lymphoblasts were further gated into CD3^+^, CD3^+^CD4^+^ and CD3^+^CD8^+^ T cells. For each stimulus, the mean number of lymphoblasts in unstimulated cultures was subtracted from the number of lymphoblasts in the stimulated cultures, thereby compensating for baseline activation of cells. The numbers of CD4^+^, CD8^+^ and activated CD108^+^ cells for each stimulus were compared for patients before and after treatment. 

To be able to make a baseline comparison regarding available cell numbers in blood between patients and controls, we calculated the numbers of total lymphocytes, CD3^+^, CD4^+^ and CD8^+^ T cells in unstimulated cultures after seven days of culture. 

To further explore the responsiveness of cells before and after treatment, we calculated the fraction of lymphoblasts responding to different stimuli. Again, unstimulated (RPMI) cultures after seven days of culture were used to set gates for total lymphocytes and lymphoblasts based on FSC and SSC properties. Using these gates, we calculated the fractions of responsive lymphoblasts for the different stimuli, expressed as percentage of lymphocytes. This was achieved by dividing the number of gated blasts by the number of total lymphocytes after seven days of culture.

### Statistics

For comparisons of flow cytometry and lymphocyte activation assay data, paired samples t-test was performed. Bi-variate correlation analyses (Pearson) were used to examine possible associations between flow cytometry and clinical variables. Flow cytometry data is given as mean values ± standard deviation (SD). For comparison between lymphocyte subpopulations at one-year follow-up, independent samples t test was used. For analyzing lymphocyte activation assay data, ANOVA with Tukey´s post-hoc test was used. Testing of activated lymphocyte fraction was analyzed with Kruskall-Wallis U test with Dunn´s post-hoc test. Due to multiple comparisons, p<0.01 was considered statistically significant and p<0.05 was considered a tendency. All statistical calculations were performed in SPSS 20.0 software (SPSS inc., Chicago, IL, USA).

## Results

### Clinical and CSF variables; changes after one year of natalizumab treatment

Although this was an observational study with the purpose of evaluating immunological effects of treatment, also clinical and CSF variables were recorded in a prospective manner. During the one-year follow-up, 34 patients were free from relapses, four patients had one relapse and two patients had two relapses. Four patients had a relapse within one month before follow-up sampling of peripheral blood, and two of these patients received treatment with methylprednisolone. The annualized relapse rate decreased from 1.0 to 0.1 on treatment. There was a significant improvement in clinical scoring systems as well as a decrease in CSF total white blood cell counts and IgG index at follow-up (Table 2).

**Table 2:** Clinical and CSF data at baseline and at follow-up after one year of natalizumab treatment.

Clinical /CSF parameters	Baseline	Follow-up	p
EDSS	2.5 (0-7.0)	2.5 (0-8.0)	0.08
MSSS	3.82 (0.19-8.55)	3.20 (0.17-9.20)	<0.0005
MSIS-29			
physical	2.18 (1.00-4.75)	1.40 (1.00-4.20) **^*a*^**	<0.0005
psychological	2.11 (1.00-4.56)	1.44 (1.00-4.56) **^*a*^**	<0.0005
SDMT	48 (5-66)	50 (11-65) **^*b*^**	0.03
Total CSF wbc count	2.55 (0.2-28.0) **^*c*^**	1.1 (0.0-4.0) **^*d*^**	<0.0005
IgG index	0.92 (0.48-3.0) **^*c*^**	0.77 (0.45-2.4) **^*d*^**	<0.0005
Albumin ratio	4.4 (2.1-11.4) **^*c*^**	4.7 (1.8-10.1) **^*d*^**	0.3

Median values are given and range within parenthesis. n=40 unless stated otherwise. p refers to Wilcoxon signed rank test comparing baseline and follow-up. ***^a^*** n=37 because of lack of follow-up data. ***^b^*** n=38 because of lack of follow-up data. ***^c^*** n=38 since two patients refrained from lumbar puncture at baseline. ***^d^*** n=36 since four patients refrained from lumbar puncture at follow-up.

Abbreviations: EDSS=Expanded Disability Status Scale, MSSS=Multiple Sclerosis Severity Score, MSIS-29=Multiple Sclerosis Impact Scale 29, SDMT=Symbol Digit Modalities Test, wbc=white blood cell, NA=not applicable

### Changes in lymphocyte populations after one year of natalizumab treatment

#### Main lymphocyte populations

**Figure 1:**
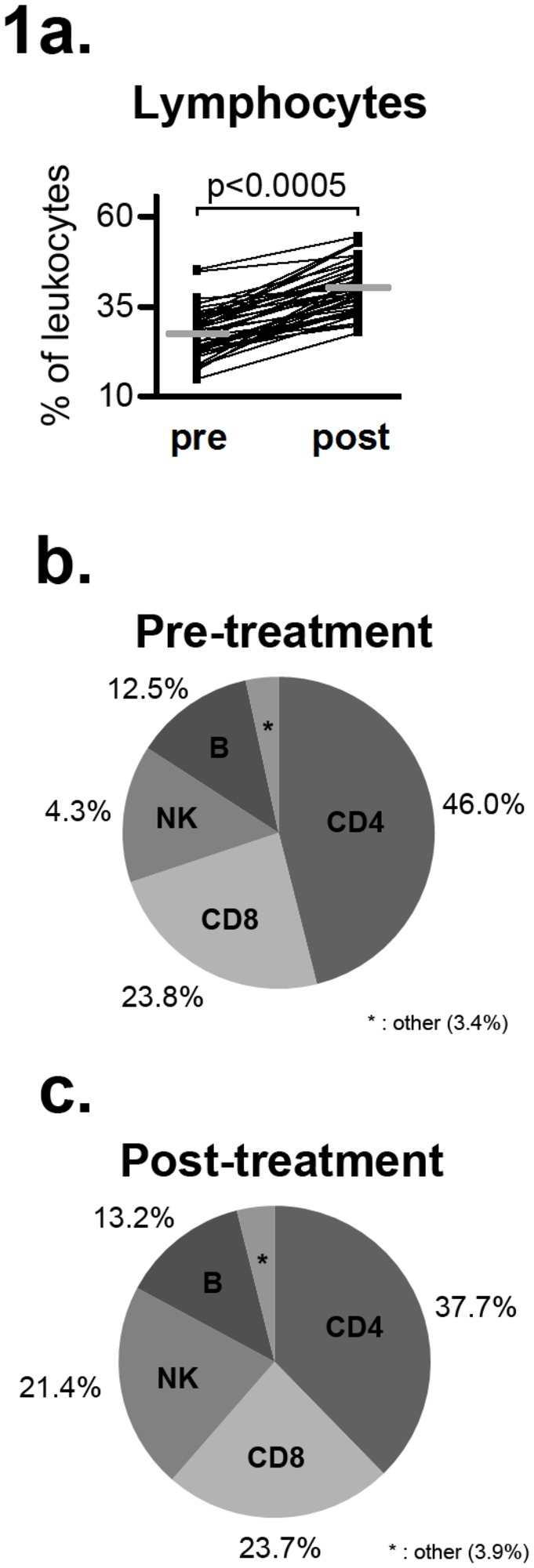
Overview of lymphocyte populations in patients before and after one year of natalizumab treatment. **a**: Distribution of lymphocytes (% of total leukocytes). Comparisons are pairwise. Bars denote mean values. **b**-**c**: Relative distribution of discrete lymphocyte subpopulations before (b) and after (c) natalizumab treatment.

Absolute numbers of all investigated lymphocyte populations were significantly increased at follow-up (Table 3). However, the relative size (percentage of parent population) of the increase differed across lymphocyte populations, leading to increased fractions of NK cells and CD8^+^ T cells, whereas fractions of CD3^+^ T cells and CD4^+^ T cells decreased (Figure 1 a-c, Table 3). 

**Table 3:** Changes in main lymphocyte populations in peripheral blood before (baseline) and after one year (follow-up) of treatment with natalizumab.

	Number (cells/μl)					Percentage of parent population (%)			
	baseline	follow-up	change	p		baseline	follow-up	change	p
Lymphocytes	1989 ± 630	3889 ±1163	+96 %	<0.0005		27.7 ± 6.8	40.3 ± 7.0	+45 %	<0.0005
T cells	1501 ± 554	2591 ± 915	+73 %	<0.0005		75.0 ± 7.0	66.1 ± 6.7	-12 %	<0.0005
CD4^+^	896 ± 285	1435 ± 397	+60 %	<0.0005		61.3 ± 9.7	56.6 ± 7.7	-8 %	<0.0005
CD8^+^	502 ± 316	975 ± 599	+94 %	<0.0005		31.8 ± 8.4	35.9 ± 8.6	+13 %	<0.0005
NK cells	277 ± 145	816 ± 248	+195 %	<0.0005		14.3 ± 6.6	21.4 ± 5.2	+50 %	<0.0005
B cells	258 ± 182	528 ± 296	+105 %	<0.0005		12.5 ± 6.4	13.2 ± 5.1	+6 %	0.3

Mean ± SD, n=40 except for CD4^+^ where n=38. p values refers to paired samples t test comparing number of cells and percentage of parent population, respectively, at baseline and follow-up. Change refers to difference between baseline and follow-up mean, given in % of baseline values.

#### CD4+ T cells

**Figure 2:**
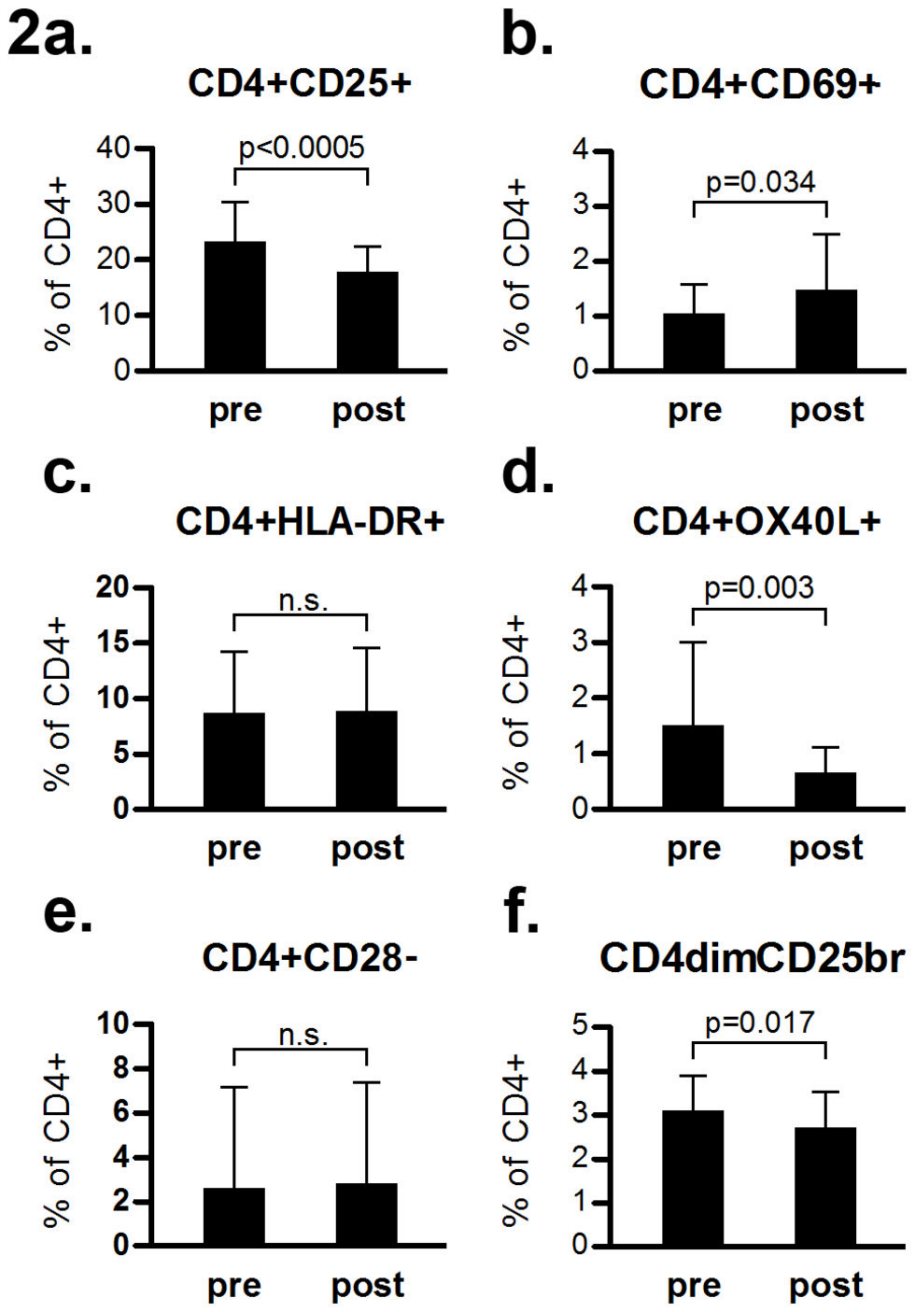
Phenotypic characteristics of CD4+ peripheral lymphocyte subpopulations in MS patients before and after one year of natalizumab treatment (pre and post, respectively). p<0.01 is considered statistically significant. Comparisons are pairwise. Bars show mean values, whiskers denote SD.

The fraction of activated T helper cells, expressed as CD4^+^CD25^+^ (including both CD25^dim^ and CD25^bright^ cells) among CD4^+^ cells, decreased (from 23 ± 7.4 to 18 ± 4.6, Figure 2 a). However, the percentage of activated T helper cells as defined by the early activation marker CD69 (CD4^+^CD69^+^ cells), tended to increase (from 1.0 ± 0.5 to 1.5 ± 1.0, Figure 2 b). Fractions of CD4^+^OX40L^+^, representing activated immunomodulatory T cells, decreased (from 1.5 ± 1.5 to 0.6 ± 0.5, Figure 2 d) and CD4^dim^CD25^bright^ Treg cells tended to decrease (from 3.1 ± 0.8 to 2.6 ± 0.8, Figure 2 f). The fractions of CD4^+^HLA-DR^+^, and CD4^+^CD28^-^ subpopulations did not change pre- to post-treatment (Figure 2 c, e).

#### CD8+ T cells

**Figure 3:**
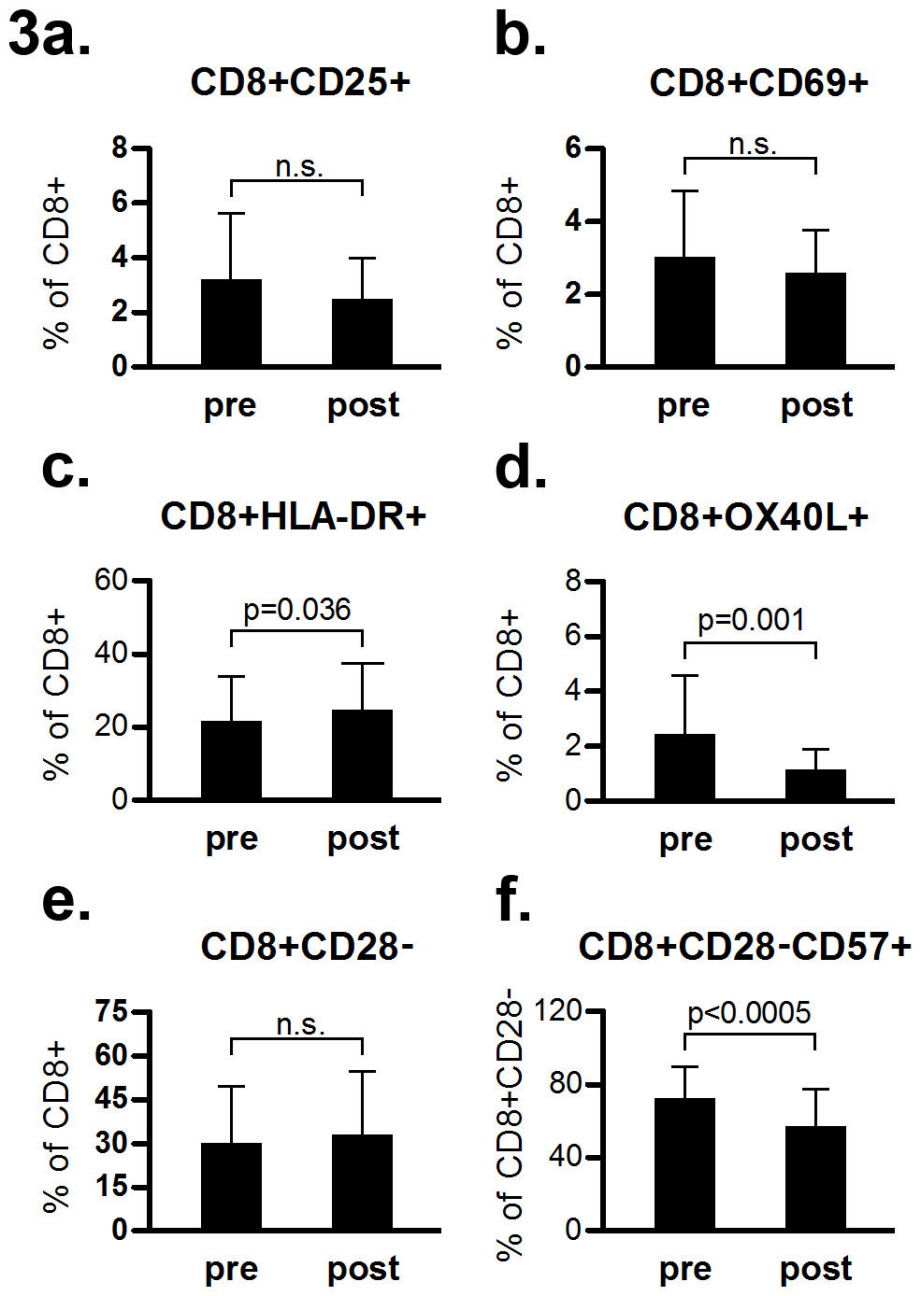
Phenotypic characteristics of CD8+ peripheral lymphocyte subpopulations in MS patients before and after one year of natalizumab treatment (pre and post, respectively). p<0.01 is considered statistically significant. Comparisons are pairwise. Bars show mean values, whiskers denote SD.

The fraction of late activated cytotoxic T cells, represented by CD8^+^HLA-DR^+^ cells among CD8^+^ cells, tended to increase (from 21 ± 12 to 24 ± 13, Figure 3 c). Fractions of CD8^+^OX40L^+^ T cells, representing activated immunomodulatory T cells, decreased from 2.4 ± 2.2 to 1.1 ± 0.8 Figure 3 d). Senescent cytotoxic CD8^+^CD28^-^CD57^+^ T cells decreased (from 72 ± 18 to 56 ± 21, Figure 3 f). The fractions of CD8^+^CD25^+^, CD8^+^CD69^+^ and CD8^+^CD28^-^ subpopulations did not change pre- to post-treatment (Figure 3 a-b, e)

#### B cells, NK and NKT cells

**Figure 4:**
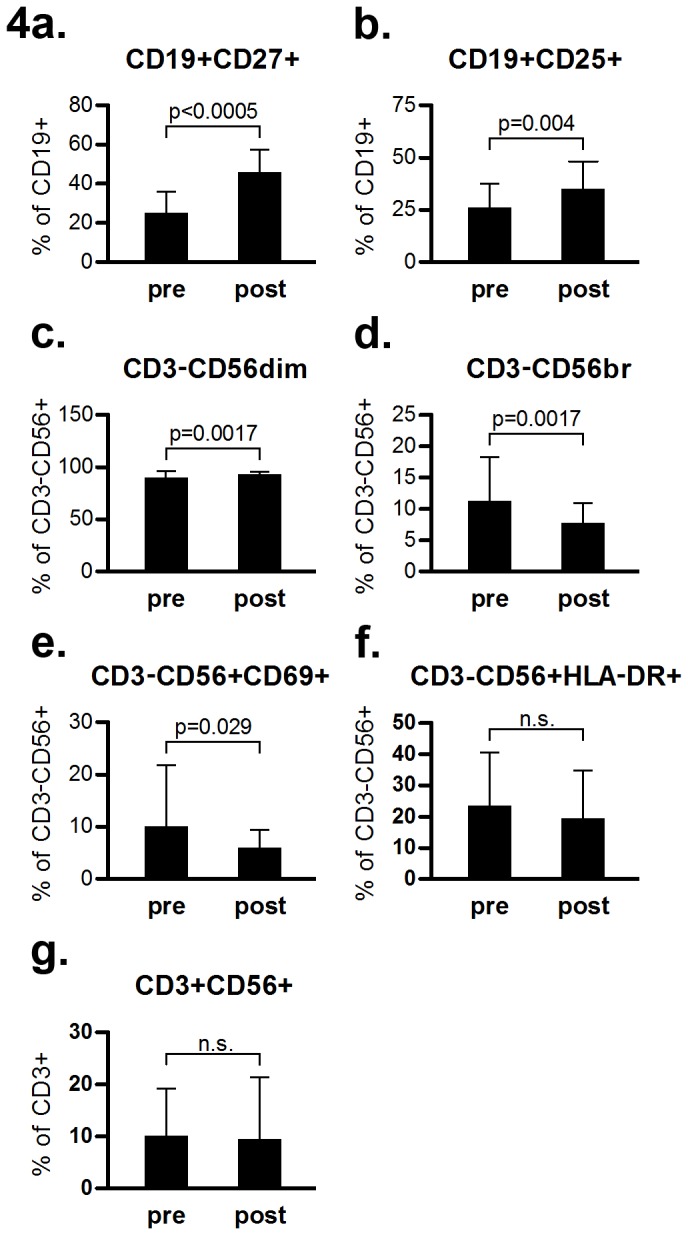
Phenotypic characteristics of CD19+ B cell and CD3^-^CD56+ NK cell subpopulations in MS patients before and after one year of natalizumab treatment (pre and post, respectively). p<0.01 is considered statistically significant. Comparisons are pairwise. Bars show mean values, whiskers denote SD.

The fraction of CD19^+^CD27^+^ cells, representing memory B cells, increased (from 25 ± 11 to 45 ± 12), as did the fraction of CD19^+^CD25^+^ cells, presumably representing regulatory B cells (Breg) (from 25 ± 12 to 35 ± 14, Figure 4 a-b). The increase in the fraction of memory B cells was higher than the increase in the Breg population (p=0.005). Among CD3^-^CD56^+^ NK cells, an increase in the percentage of CD3^-^CD56^dim^ NK cells (from 89 ± 7.2 to 92 ± 3.3, Figure 4 c) was accompanied by a decrease in CD3^-^CD56^bright^ NK cells (from 11.1 ± 7.2 to 7.6 ±3.3, Figure 4 d). Early activated CD3^-^CD56^+^CD69^+^ NK cells tended to decrease (from 9.9 ± 12 to 5.8 ± 3.6, Figure 4 e). The percentages of CD3^-^CD56^+^HLA-DR^+^and total CD3^+^CD56^+^ NKT cells did not change pre-to post-treatment (Figure 4 f-g), as was also true for percentages of total NK cells and CD4^+^CD56^+^ NKT and CD8^+^CD56^+^ NKT subpopulations (data not shown).

### Lymphocyte activation assay

**Figure 5:**
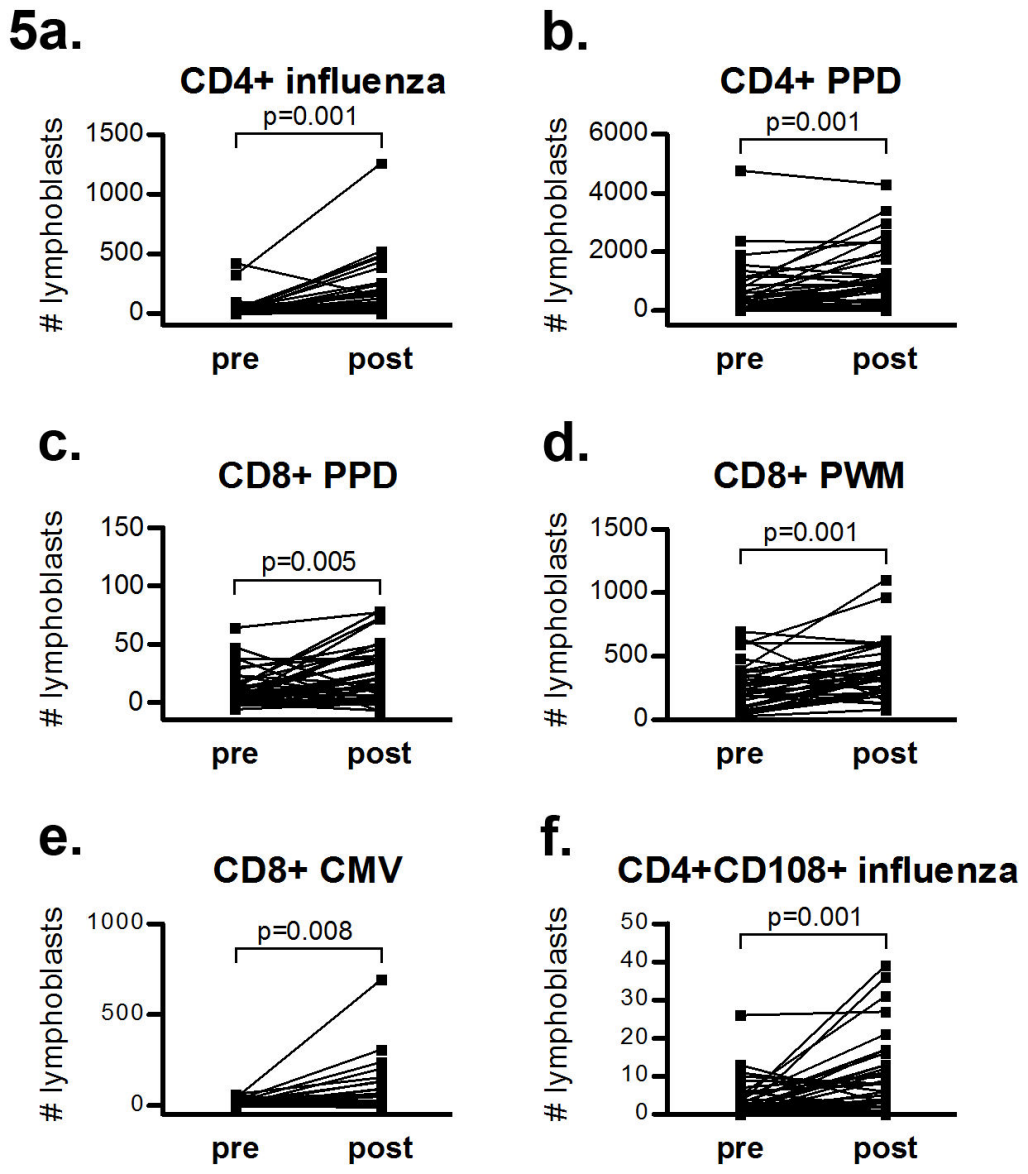
Lymphocyte activation responses towards antigens and mitogens in different T cell populations. Pairwise comparisons between patients before and after one year of natalizumab treatment. n=37 in both groups.

The number of influenza antigen-activated CD4^+^ lymphoblasts increased after treatment (from 39 ± 85 cells/μl to 170 ± 236 cells/μl), as did the PPD-activated lymphoblasts (from 595 ± 914 to 1060 ± 1043, Figure 5 a-b). In addition, CD4 responses to both PWM (p<0.05) and CMV (p<0.05) tended to increase (data not shown). For CD8^+^ cells, an increased response was observed upon stimulation with PPD (from 11 ± 16 to 23 ± 25), PWM (from 262 ± 180 to 385 ± 215) and CMV (from 10 ± 17 to 68 ± 130, Figure 5 c-e). CD4^+^CD108^+^ activated T helper cells showed a stronger response towards influenza antigen post-treatment (from 3.6 ± 5.1 to 9.7 ± 10, Figure 5 f). Furthermore, MBP-induced responses tended to increase among CD4^+^CD108^+^ cells in treated patients (from 0.2 ± 0.7 to 0.7 ± 1.2, p=0.034). A similar tendency was seen in CD8^+^CD108^+^ activated cytotoxic cells after influenza stimulation (0.2 ± 0.7 to 0.8 ± 1.4, p=0.013).

**Figure 6:**
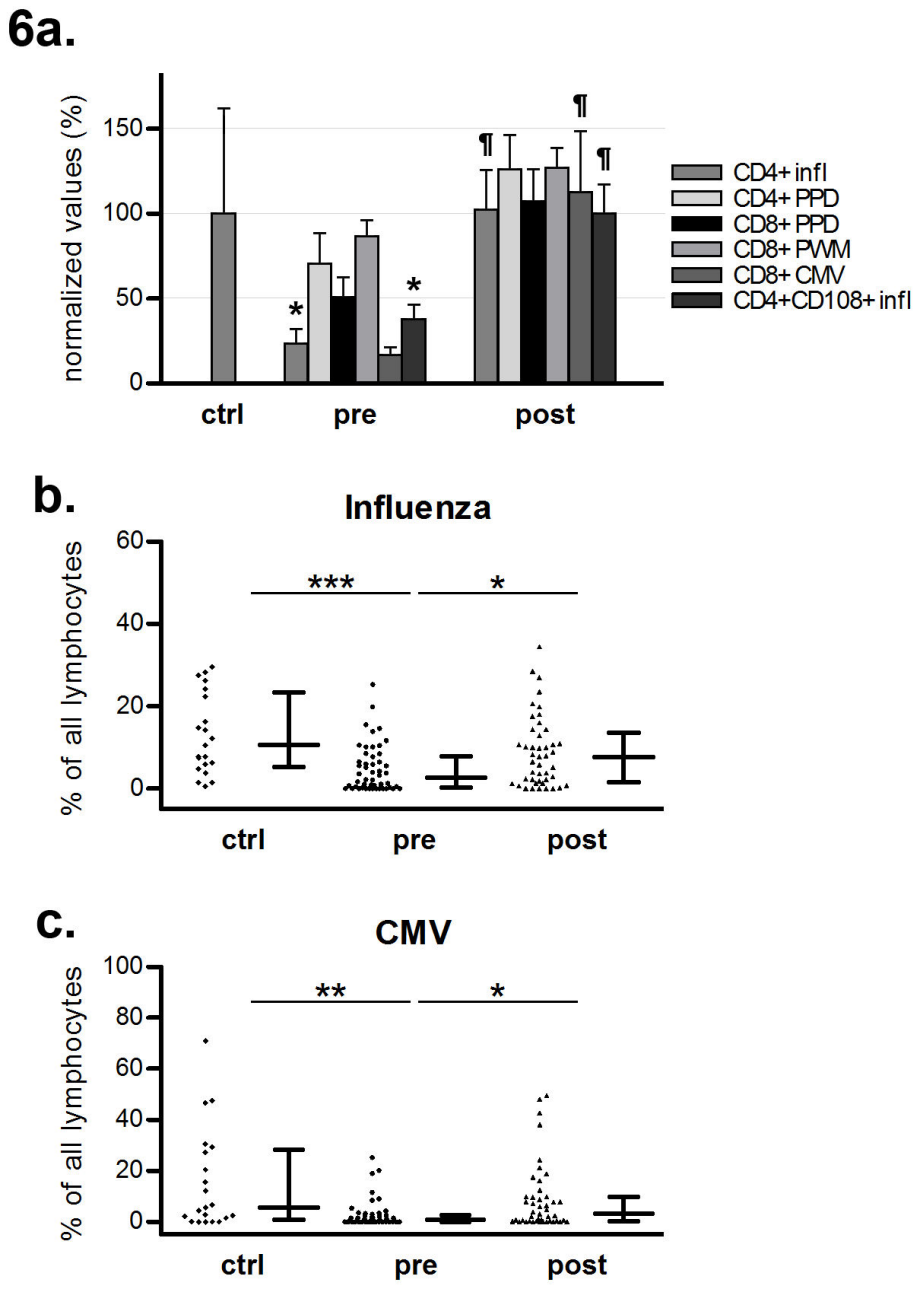
Lymphocyte activation responses in patients and controls. **a**: Responses towards antigens and mitogens in healthy controls and patients before and after one year of natalizumab treatment. For visualization purposes, data are normalized to the average of the healthy controls for the respective antigens. Analysis performed with one-way ANOVA with Tukey´s post hoc test. * p<0.05, comparison between controls and pre-treatment patients. ¶ p<0.05, comparison between pre- and post-treatment patients. n=23 for controls, n=37 for both pre- and post-treatment groups. Bars show mean values, whiskers denote SEM. **b**-**c**: Fraction of activated lymphocytes in response to influenza (b) and CMV (c). Groups compared with Kruskall-Wallis test, utilizing Dunn´s post-hoc test. Median and interquartile range are shown. * p<0.05, ** p<0.01, *** p<0.005.

As controls, we analyzed the response of cell cultures in peripheral blood of healthy individuals. We found a stronger response in healthy individuals compared with pre-treatment levels of patients regarding influenza antigen-induced CD4^+^ and CD4^+^CD108^+^ T cell responses (166 ± 247 versus 39 ± 85 and 9.7 ± 14 versus 3.6 ± 5.1, respectively, Figure 6 a). However, post-treatment levels of patients were in the same range as those for controls. Since the FASCIA method used for these analyses takes into account both the total number of cells in the culture, as well as the responsiveness of these cells, we wanted to further explore the nature of the decreased response observed in pre-treatment patients compared to that of healthy controls. Analysis of unstimulated (RPMI) cultures revealed that numbers of cells were comparable between pre-treatment patients and controls, while post-treatment patients exhibited significantly increased cell numbers compared to pre-treatment patients, as well as compared to controls (see Figure S1 a-d). This finding implies that the decreased responsiveness in pre-treatment patients is not dependent on a low number of cells in culture and indicates that the increase in cell numbers post-treatment has a major impact on the increase in responsiveness.

Furthermore, to evaluate the function on a cell-by-cell basis, the fraction of lymphoblasts responsive to stimuli (expressed as proportion (%) of lymphoblasts out of lymphocytes) revealed that the fraction of cells responding to Influenza antigen and CMV were lower in pre-treatment patients compared to controls (p<0.005 for Influenza, p<0.01 for CMV, Figure 6 b-c). The fractions of stimulated cells tended to increase in post-treatment compared with pre-treatment samples (p<0.05 for both Influenza and CMV, Figure 6 b-c), and post-treatment there was no difference between patient and controls. Thus, the increased responsiveness seen in patients post-treatment might in part be attributable to a component of increased per-cell responsiveness, in addition to increased lymphocyte cell numbers.

### Lymphocyte population composition versus clinical variables

No associations were found between pre-treatment peripheral blood composition of major lymphocyte population numbers (total lymphocytes, T, B and NK cells, CD4^+^ and CD8^+^ cells) or subpopulation fractions of lymphocytes (CD3^+^CD56^+^ NKT, CD3^-^CD56^bright^ and CD3^-^CD56^dim^ NK cells, CD4^dim^CD25^bright^ Treg cells, CD19^+^CD27^+^ memory B cells, CD19^+^CD25^+^ Breg cells) versus pre-treatment clinical data (disease duration, number of relapses last two years, MSSS) (data not shown). Furthermore, no associations were found between one-year change in different lymphocyte population numbers (total lymphocytes, CD3^+^, CD4^+^, CD8^+^, CD3^-^CD19^+^, CD3^-^CD56^+^) or subpopulation fractions of T cells (CD3^+^CD56^+^, CD3^-^CD56^bright^, CD3^-^CD56^dim^, CD4^+^CD25^bright^, CD19^+^CD27^+^, CD19^+^CD25^+^) versus number of relapses during one-year treatment (data not shown).

## Discussion

In this one-year longitudinal observational study, by assessing changes in circulating lymphocyte compositions of 40 patients with relapsing MS treated with natalizumab, we demonstrated a significant increase in absolute numbers of all major populations, most markedly for NK cells and B cells, as well as restored CD4^+^ and CD8^+^ T cell responsiveness to recall antigens and mitogens. 

An increase in numbers of circulating lymphocytes during natalizumab treatment corroborates with the natalizumab´s blocking effect on lymphocyte extravasation [5]. The parallel findings of a reduction in leukocyte counts and IgG index intrathecally also support the blocking effect of natalizumab on CNS cell-trafficking [6,7]. VLA-4 (α4β1) is widely expressed on many different lymphocyte populations including T cells, B cells, NK cells as well as on a majority of monocytes and macrophages. The interaction of VLA-4 and its ligands is not specific for the blood-brain barrier (BBB) since VCAM-1 is expressed on activated endothelium throughout the body [26]. Thus, the overall effect of VLA-4 interference on lymphocyte populations measured in blood is not only a result of reduced migration across the BBB but also across endothelium in other peripheral tissues. Furthermore, considering the low numbers of lymphocytes intrathecally compared with numbers in the periphery, it is unlikely that reduced migration to the CNS may account for the total increase in circulating lymphocytes during natalizumab treatment. In addition, it was found that natalizumab mobilizes hematopoietic progenitor cells out of the bone marrow [27]. Also, besides effects on cell migration, a co-stimulatory effect on VLA-4 by natalizumab, may also contribute to changes in circulating lymphocyte subsets [28].

VLA-4 expression varies between different lymphocyte populations, being higher on B cells than on T cells, and higher on CD8^+^ than on CD4^+^ T cells [13]. Furthermore, the amount of natalizumab binding to CD3^-^ NK cells may be higher than the amount binding to CD19^+^ B cells and CD3^+^ T cells in a descendant scale [29]. This diversity in binding preference of natalizumab is well in line with our observation of the highest increase in the number of NK cells after treatment (195% increase compared with baseline), followed by B cells (105% increase compared with baseline) and T cells (73% increase compared with baseline).

NK cells are part of the innate immune system and have both cytotoxic and regulatory properties [30]. Even though NK cells were observed to have the most pronounced relative increase of all circulating lymphocytes, the fraction of regulatory NK cells (CD3^-^ CD56^bright^) decreased with a concomitant increase in cytotoxic NK cells (CD3^-^ CD56^dim^). Based on different expression of chemokine receptors and adhesion molecules, cytotoxic NK cells and regulatory NK cells have different migration preferences with CD56^dim^ migrating to inflammatory sites while CD56^bright^ preferentially home to secondary lymphoid organs [31]. Our novel finding of an increase in the fraction of cytotoxic NK cells in blood after treatment seems logic when considering these cells preference for homing to inflammatory sites but now sequestered in the circulation due to natalizumab. Since VCAM-1 is up-regulated at sites of inflammation, cytotoxic NK cells may be relatively more affected by the blocking effect on VLA-4 compared with regulatory NK cells. 

The OX40-OX40L interaction has been ascribed an important role in promoting survival and clonal expansion of effector and memory T cells, regulating T cell-mediated cytokine production [32] and facilitating Th2 immune responses [33]. In the murine model of MS, experimental allergic encephalomyelitis (EAE), blocking the OX40-OX40L interaction ameliorated the disease [34]. We here report a significant decline in proportions of both CD4^+^OX40L^+^ and CD8^+^OX40L^+^ cells after natalizumab treatment, indicating an attenuation of effector T cell responses in the periphery. Since OX40-OX40L interactions favor Th2 immune responses [33], our result may also have implications on the Th1-Th2 balance systemically. 

Absolute numbers of B cells also increased after treatment. Interestingly, the fractions of both memory B cells (CD19^+^CD27^+^) and presumed Breg cells (CD19^+^CD25^+^) [35] increased. In contrast to naïve B cells (CD19^+^CD27^-^), memory B cells secrete the pro-inflammatory cytokines tumor necrosis factor (TNF) and lymphotoxin (LT) upon stimulation [36], and Breg cells suppress CD4^+^ T cell proliferation and enhance Treg cell properties [37]. The increase was higher in memory B cells than in presumed Breg cells, which may have implications for B cell responses both in the periphery and in the CNS. The increase in the fraction of memory B cells is in accordance with recent reports where an increase in memory B cells was observed while the population of naïve B cells decreased [17,38]. The latter finding was suggested to depend on differences in α4-integrin expression between these two B cell subsets. Taken together, the marked increase in circulating B cells during natalizumab treatment is a consistent finding throughout many studies and further yields this lymphocyte population a probably essential role in treatment effects and side effects.

In addition to the profound changes in lymphocyte populations, we observed increased CD4^+^ and CD8^+^ T cell responses to recall antigens and mitogens in whole blood during treatment. Although different subpopulations of T cells showed some variations in responses to different antigens, the overall pattern was consistent with higher responses post-treatment. Prevention of immune cell entry into tissues by natalizumab may lead to an increase in the number of reactive memory cells in the circulation, in accordance with the observation by Börnsen et al. [16], for T cells and our present observation for B cells. Furthermore, the overall reduction in T cell responsiveness noted in pre-treatment MS patients compared with healthy controls, was restored by natalizumab treatment. 

The FASCIA method that we used reckons both the number and function of cells, thus providing a measure of the total functional capacity, which indeed is of clinical relevance. A detailed analysis taking into account both total numbers of cells available in the sample and the proportion of cells responding to stimuli showed that the increase in responsiveness observed after natalizumab treatment to a large extent was explained by an increase in cell numbers but there was also an increase in function on a cell-by-cell basis. This finding is not in full agreement with the finding of Börnsen et al [16], since they found no functional difference between untreated and natalizumab-treated MS patients regarding responses to tetanus and MBP. This discrepancy may be explained by material selection, as for example the untreated group [16] comprised mainly early-phase MS patients. Although our finding of lowered T cell responsiveness in MS patients compared with controls was not included as an aim of the study, it is an interesting observation that is in line with some previous literature indicating a defect responsiveness in MS for example to anti-CD3 stimulation [39] to PWM [40] as well as to stimulation by viral antigens [40,41]. However, our finding of lowered responsiveness in MS patients compared to controls may also depend on treatment prior inclusion, since 30 patients out of 40 received immunomodulating treatment within four months before baseline.

In conclusion, our findings indicate a preserved or increased ability for immune responses systemically after one-year natalizumab treatment. However, since natalizumab reduces lymphocyte extravasation not only to the CNS, immune surveillance and responses in peripheral tissues may be insufficient, which should be accounted for in treatment considerations. The systemic increase in the major populations (NK cells more than B cells more than T cells, respectively) may be explained by expression levels of VLA-4 on these populations, thus reflecting the effect of natalizumab on cell trafficking. However, the differential effects on subsets of these populations, including markers of activation and co-stimulation, are unlikely to be explained by cell traffic effects alone, indicating additional effects of natalizumab involving also cell-signalling.

## Supporting Information

Figure S1 **Absolute number of unstimulated cells after 7 days of culturing.** Mean and SD values are shown. Differences shown mark comparisons between pre- and post-treatment patients, and for post-treatment patients and controls, respectively. All comparisons were made using one-way ANOVA with Tukey’s post-hoc test. No significant differences were observed when comparing pre-treatment patients and controls. **: p<0.01, ***: p<0.005.
(TIF)
