# Supplementary figures and images for: Increased B Cell and Cytotoxic NK Cell Proportions and Increased T Cell Responsiveness in Blood of Natalizumab-Treated Multiple Sclerosis Patients
Source: PLoS One. 2013 Dec 2;8(12):e81685. doi: 10.1371/journal.pone.0081685 (PMC3847051; doi:10.1371/journal.pone.0081685)

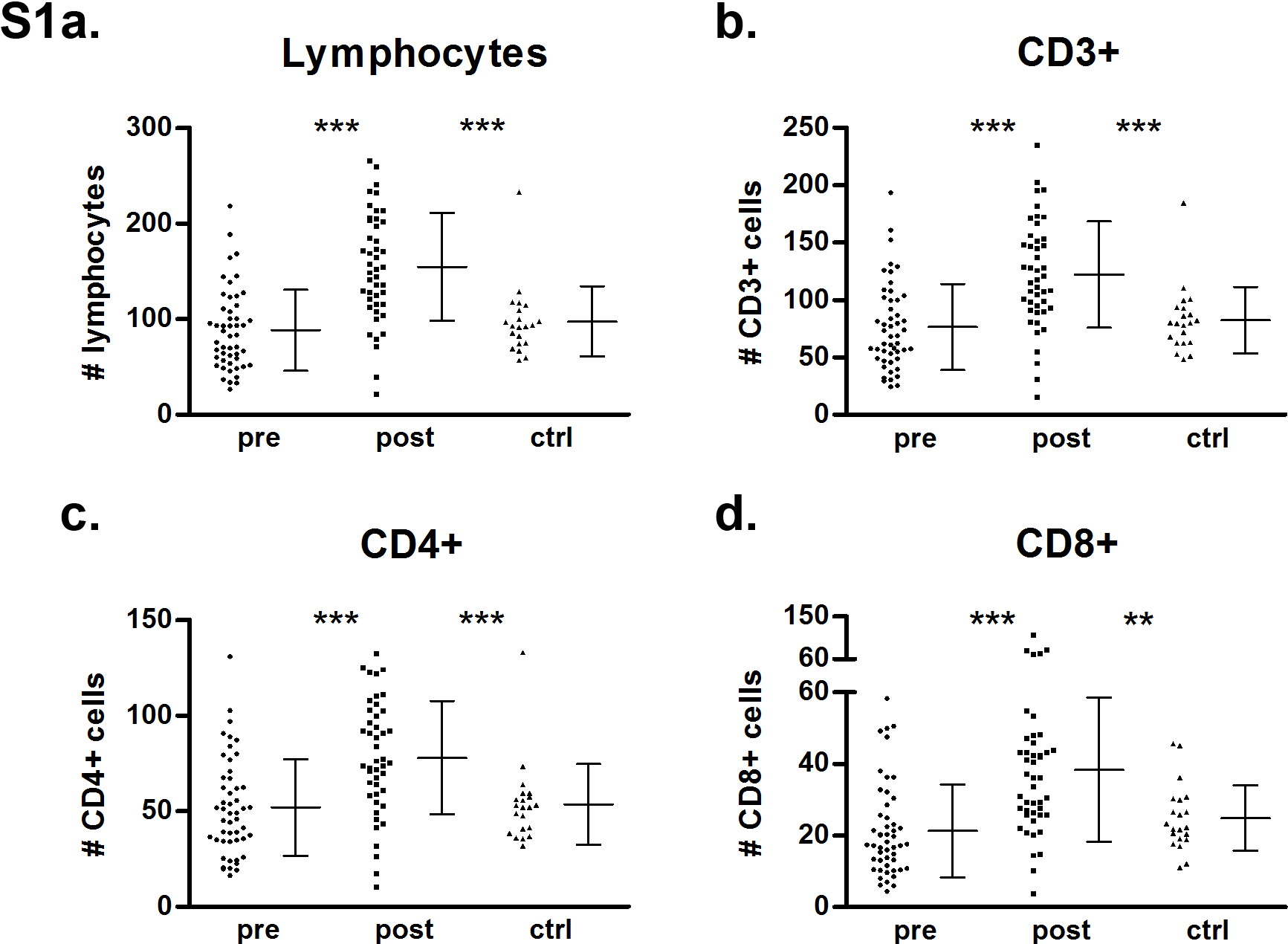

Supplement: Figure S1 — Absolute number of unstimulated cells after 7 days of culturing. Mean and SD values are shown. Differences shown mark comparisons between pre- and post-treatment patients, and for post-treatment patients and controls, respectively. All comparisons were made using one-way ANOVA with Tukey’s post-hoc test. No significant differences were observed when comparing pre-treatment patients and controls. **: p<0.01, ***: p<0.005. (TIF) [file pone.0081685.s001.tif]
